# Quantitative examination of the inhibitory activation of molecular targeting agents in hepatocellular carcinoma patient‐derived cell invasion via a novel in vivo tumor model

**DOI:** 10.1002/ame2.12085

**Published:** 2019-09-27

**Authors:** Huiwei Sun, Fan Feng, Hui Xie, Xiaojuan Li, Qiyu Jiang, Yantao Chai, Zhijie Wang, Ruichuang Yang, Ruisheng Li, Jun Hou

**Affiliations:** ^1^ Research Center for Clinical and Translational Medicine The Fifth Medical Center General Hospital of Chinese PLA Beijing China; ^2^ Center for Clinical Laboratory The Fifth Medical Center General Hospital of Chinese PLA Beijing China; ^3^ Department of Interventional Therapy The Fifth Medical Center General Hospital of Chinese PLA Beijing China; ^4^ Medical School of Chinese PLA Beijing China

**Keywords:** hepatocellular carcinoma, in vivo invasion, molecular targeting agents, patient‐derived cells

## Abstract

**Background:**

The outcomes for patients with advanced hepatocellular carcinoma (HCC) receiving sorafenib are far from satisfactory because of treatment resistance to sorafenib. However, the exact mechanism of resistance to sorafenib remains unclear and it is valuable to establish a novel mouse model to quantitatively analyze the inhibition rates of sorafenib on the invasive growth of HCC cells in the liver.

**Methods:**

HCC tissue microblocks derived from patients were cultured and mixed with hydrogel drops. Then, hydrogel drops containing microblocks of HCC tissue were attached onto the surface of the livers of nude mice to form lesions or nodules of HCC. The mice received molecular targeting agents through oral administration. Livers with tumor nodules were harvested for H&E staining (hematoxylin‐eosin staining) analysis and H&E staining images were quantitatively analyzed using image J software. The invasive growth of HCC cells into the liver was calculated using the depth of the lesions compared with the total thickness of the liver.

**Results:**

Microblocks containing cells derived from HCC patients can form lesions in the liver of nude mice. Oral administration of molecular targeting agents inhibited the invasive growth of HCC cells in the liver of nude mice.

**Conclusions:**

The model established in this study involves the invasive growth of HCC cells in the liver of nude mice, and the model allows for the quantitative analysis of the inhibitory effect of molecular targeting agents on the invasion of HCC cells in vivo.

## 1 INTRODUCTION

Hepatocellular carcinoma (HCC) is still one of the foremost public health threats in Asia and the Pacific region, and specifically in China, because of high rates of hepatitis virus, such as hepatitis B virus (HBV).^1^, ^2^ Moreover, a large proportion of patients are diagnosed at the advanced stage of HCC at initial diagnosis and cannot receive certain treatments, such as surgery.^3^ Advanced HCC is also known for its multi‐drug resistance (MDR) features, whereby it is not sensitive to cytotoxic chemotherapies.^4^ Therefore, current antitumor agents for advanced HCC are molecular targeting agents, represented by sorafenib.^5^ However, during clinical treatment, some patients are initially resistant to sorafenib (initial resistance) and other patients who are initially sensitive to sorafenib can acquire resistance to sorafenib.^6^ Therefore, it is valuable to determine whether a patient is sensitive to molecularly targeted drugs or if they are suitable to receive molecular targeting agents.

The patient‐derived tumor xenograft (PDX) animal model involves inoculating patient‐derived tumor cells into nude mice for an investigation of drug antitumor activity.^7^ The PDX model is also an in vivo study model that can be used to detect the antitumor activity of drugs in animals. However, patient‐derived tumor cells can reflect the sensitivity of cells in patients' tumor tissues to antitumor drugs.^8^ In this work, patient‐derived HCC tissues were prepared as tumor tissue microblocks, and a medical hydrogel was used to adhere the tissue microblocks to the liver surface in nude mice. A quantitative study of the invasive growth of HCC cells was performed by measuring the invasive depth of the cells in nude mice, and the effect of the oral administration of molecularly targeted drugs on HCC was measured by calculating the inhibition rate of HCC cell invasive growth. The sensitivity of the cells to molecularly targeted drugs was also assessed.

## 2 MATERIALS AND METHODS

### 2.1 Tumor tissues

The collection of the clinical tissues and the study protocol were approved by the Ethics Committee of the fifth Medical Center of the Chinese People's Liberation Army General Hospital (original name: the 302nd Hospital), and written consent was obtained from all patients. The clinical tissues were obtained through a puncture biopsy using a coaxial needle (cat. no.: MCXS1815BP, RITA Company) immediately before radiofrequency ablation treatment, following a method described in our previous work.^9^ The obtained tissues were preserved using Dulbecco's Modified Eagle Medium (DMEM) supplemented with 20% fetal bovine serum (FBS), and tumor tissue microblocks were prepared on a clean bench. The weights of the tumor tissue microblocks were accurately measured using a precision balance, and the size of the tumor microblocks was adjusted to make the weight of the microblocks uniform, at approximately 1 mg per microblock.

### 2.2 Preparation of oral‐administration formulations of the molecular targeting agents

Molecular targeting agents were purchased from Selleck Corporation (Houston, TX, USA): sorafenib (cat. no.: S7397), regorafenib (cat. no.: S1178), lenvatinib (cat. no.: S1164), anlotinib (cat. no.: S8726) or apatinib (cat. no.: S5248). Molecular targeting agents' oral‐administration formulations were prepared referring to a method described by Wu et al^10^ and Wang et al^11^ Briefly, the drug powders were accurately weighed and dissolved with organic solvent dimethyl sulfoxide (DMSO), polyethylene glycol (PEG) and Tween 80 to prepare the drug solution. Next, the drug solution was diluted by physiological saline, assisting with sonication or stirring during the dilution process. The concentration of the organic solvents DMSO, PEG and Tween 80 in the final preparation did not exceed 1‰, 2‰ and 2‰. The molecular targeting agents' oral‐administration formulations were packed and preserved at −80℃.

### 2.3 MicroPET screening (living‐imaging analysis of nude mice)

All animal experiments in thise present work wereh been reviewed and approved by the Animal Care and Usage Committee of the fifth Medical Center of the Chinese People's Liberation Army General Hospital (original name: the 302nd Hospital), and the studies were performed in accordance with the UK Animals (Scientific Procedures) Act of 1986 and the associated guidelines. The animal experiments were all performed in accordance with the ARRIVE guidelines (Animal Research: Reporting of In vivo Experiments). Nude mice (BALB/c with thymus deficiency) that were 4‐6 weeks old were purchased from Si‐Bei‐Fu Corporation, Beijing, China and were used in this work. The ethic‐approval number of the project is IACUC‐2017‐013.

Firstly, inhalation anesthesia was performed on the nude mice using isoflurane as an anesthetic agent (the dose for the first anesthesia was 1.5% [volume/volume]; the dose for continuous anesthesia was 0.5% [volume/volume]). Then, the mice received a 200 μCi (7.4 MBq) dose of ^18^F‐fludeoxyglucose (FDG) via tail vein injection. After the injection (40‐50 minutes), the mice underwent microPET imaging and the microPET images were quantitatively analyzed using Image j software (NIH). The results are shown as the intensity of the tumor regions or the areas of the tumor regions in the microPET images. The inhibition rate of the molecular targeting agents was calculated as: {([intensity × total area] of the control group microPET images) − ([intensity × total area] of the administration group microPET images)}/([intensity × total area] of the control group microPET images) × 100%.^12^, ^13^

### 2.4 In vivo antitumor activation of molecular targeting agents

For subcutaneous tumor formation, inhalation anesthesia was performed on nude mice using isoflurane as an anesthetic agent (the dose for the first anesthesia was 1.5% [volume/volume]; the dose for continuous anesthesia was 0.5% [volume/volume]). Next, microblocks of HCC tissues were directly injected subcutaneously. After injection (4‐5 days), the mice received molecular targeting agents via oral administration. Mice were treated once every 2 days for a total of 10 treatments (approximately 21 days). Tissues were harvested and the tumor volumes or tumor weights of the subcutaneous tumor tissues were examined. The tumor volume was calculated as: length × width ×width/2. The tumor weights were measured using a precision balance. The inhibition rates of the molecular targeting agents were calculated as: [(tumor volume of control group tumor tissues) − (tumor volume of administration group tumor tissues)]/(tumor volume of control group tumor tissues) × 100% or [(tumor volume of control group tumor tissues) − (tumor volume of administration group tumor tissues)]/(tumor volume of control group tumor tissues) × 100%.^14^, ^15^

For an examination of intrahepatic growth, inhalation anesthesia was performed using isoflurane as an anesthetic agent (the dose for the first anesthesia was 1.5% [volume/ volume]; the dose for continuous anesthesia was 0.5% [volume/ volume]). The microblocks of the HCC clinical specimens were directly seeded into the nude mouse livers. After injection (4‐5 days), mice received molecular targeting agents via oral administration. The mice were treated once every 2 days for a total of 10 treatments (approximately 21 days). Then, the mice were examined using microPET screening. After microPET screening, tissues were harvested and the livers with lesions formed by HCC cells were collected. Images of the livers were quantitatively analyzed. The areas of the lesions were calculated as: (total pixel of lesions)/(total pixel of livers) × 100%. The inhibition rates of the agents on the intrahepatic growth of HCC cells were calculated as: {[(control group area of lesion) − (administration group area of lesion)]/(control group area of lesion)} × 100%.^12^, ^13^

**Figure 1:**
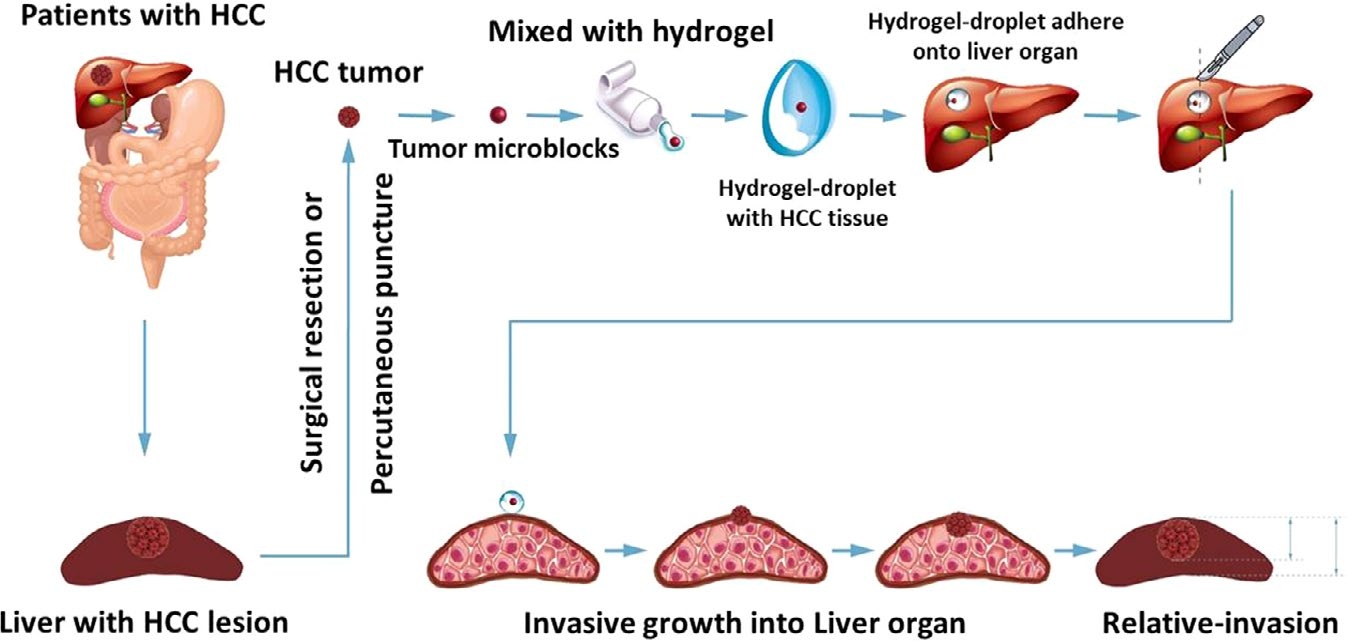
The diagram of animal model in this work

For an examination of intrahepatic invasion (Figure 1), inhalation anesthesia was performed using isoflurane as an anesthetic agent (the dose for the first anesthesia was 1.5% [volume/volume]; the dose for continuous anesthesia was 0.5% [volume/volume]). The microblocks were packaged in biological‐medical gel (Cai‐Hong‐Yi‐Xue‐She‐Bei Corporation) and then were attached onto the surface of the nude mouse livers. After injection (4‐5 days), the mice received molecular targeting agents via oral administration. The mice were treated once every 2 days for a total of 10 treatments (approximately 21 days). Then, the mice were examined using microPET screening. After microPET screening, tissues were harvested and the livers were collected for H&E staining. The tumor nodules (lesions) formed by the HCC cells were quantitatively analyzed using Image j software (NIH, USA). The thickness of the nodules formed by the HCC cells or the whole liver was examined in H&E staining images. Then, the relative invasive growth of the HCC cells into the livers was calculated based on the thickness of the nodules formed by the HCC cells or the whole livers: (the nodule thickness relative to that of the liver organ) was calculated as follows: (the lesion thickness of the control group)/(the whole liver thickness of the control group) × 100% or (the lesion thickness of the administration group)/(the whole liver thickness of the administration group) × 100%. The inhibition rate of the molecular targeting agents was calculated as follows: ([the relative invasion of the control group) − (the relative invasion of the administration group])/(the relative invasion of the control group) × 100%.

### 2.5 Statistical analysis

The statistical analysis was performed using GraphPad Software (version 6.0, San Diego, CA. USA). The difference between the two groups was assessed using two‐way analysis of variance (ANOVA) with Bonferroni correction method. A *P*‐value of less than .05 was considered to indicate statistical significance.

## 3 RESULTS

### 3.1 Molecular targeting agents inhibit the subcutaneous growth of HCC cells

**Figure 2:**
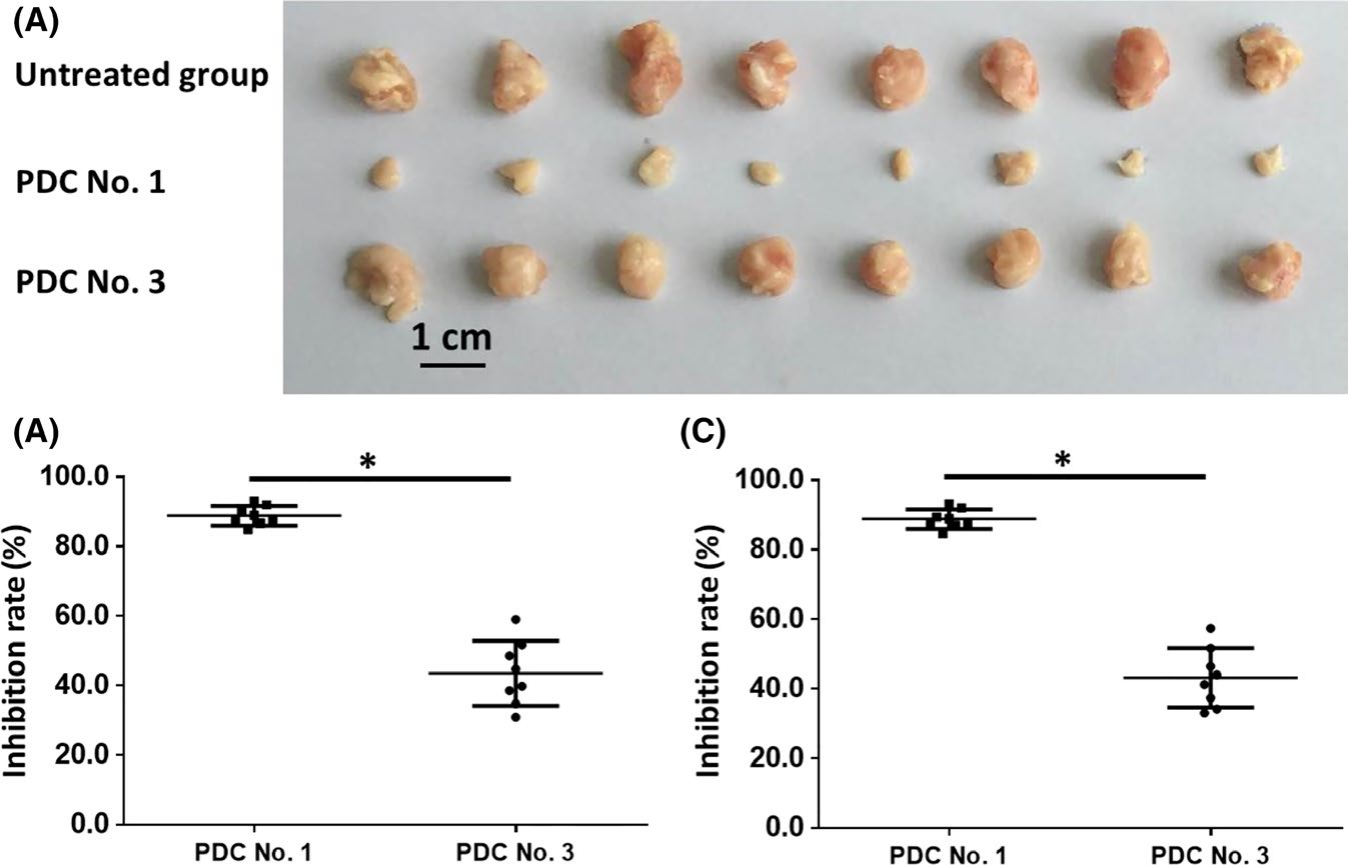
The antitumor effect of sorafenib on patient‐derived HCC cell lines. Patient‐derived tissues containing HCC cells were injected into nude mice to form subcutaneous tumors. The mice received 2 mg/kg dose of sorafenib via oral administration. Then, tumor tissues were collected. The results are shown as images of subcutaneous tumors (A) or inhibition rates of sorafenib on HCC cells based on tumor volumes (B) or tumor weights (C). *P < .05

The effect of molecular targeting agents on the subcutaneous growth of patient‐derived HCC cells was examined. As shown in Figure 2 and Figure S1, the subcutaneous transplantation in nude mice of HCC tumor tissue microblocks derived from patients resulted in tumor tissue formation. Oral administration of molecular targeting agents inhibited the subcutaneous growth of HCC cells in nude mice (Figure 2, Tables 1 and 2). The effect of sorafenib, a typical molecular targeting agent, is shown in Figure 1 as a representative result and patient‐derived cells (PDC) No. 1 and PDC No. 3 had different sensitivities to sorafenib (Figure 2). The inhibition rates of the molecular targeting agents, sorafenib, regorafenib, lenvatinib, anlotinib or, apatinib in the five PDCs are shown in Table 1 (tumor volumes) and Table 2 (tumor weights). The results showed that among these molecularly targeted agents, lenvatinib had significantly higher antitumor activity against the subcutaneous growth of the HCC PDCs compared to the other four agents.

**Table 1:** Inhibition rates of agents on HCC cells' subcutaneous tumor volume

PDCs	No. 1	No. 2	No. 3	No. 4	No. 5
	Inhibition rates on subcutaneous tumor volumes (mean ± SD, %)				
Sorafenib	88.75 ± 2.82	63.33 ± 2.15	43.48 ± 9.32	55.82 ± 3.61	50.79 ± 3.61
Regorafenib	82.53 ± 3.30	57.22 ± 3.57	33.79 ± 4.93	43.87 ± 2.79	59.38 ± 3.58
Lenvatinib	90.45 ± 2.40	71.30 ± 0.75	62.33 ± 1.52	54.62 ± 2.27	70.88 ± 2.53
Anlotinib	61.21 ± 3.35	55.23 ± 2.38	29.99 ± 3.90	43.19 ± 5.16	42.31 ± 4.20
Apatinib	69.24 ± 3.42	62.40 ± 4.20	54.14 ± 3.10	61.64 ± 3.41	57.22 ± 4.53

Abbreviation: PDCs, patient‐derived HCC cell lines.

**Table 2:** Inhibition rates of agents on HCC cells' subcutaneous tumor weights

PDCs	No. 1	No. 2	No. 3	No. 4	No. 5
	Inhibition rates on subcutaneous tumor weights (mean ± SD, %)				
Sorafenib	88.75 ± 2.82	65.24 ± 2.54	43.48 ± 9.32	58.23 ± 4.86	52.21 ± 5.02
Regorafenib	84.10 ± 2.23	55.60 ± 2.84	32.70 ± 3.77	44.03 ± 3.22	61.59 ± 4.18
Lenvatinib	89.45 ± 2.09	72.41 ± 1.32	60.13 ± 1.19	54.80 ± 1.74	71.60 ± 2.77
Anlotinib	61.44 ± 3.12	55.14 ± 2.40	33.22 ± 3.74	42.30 ± 3.24	45.78 ± 5.55
Apatinib	67.56 ± 3.59	64.40 ± 3.25	56.54 ± 2.67	64.70 ± 2.60	51.41 ± 3.61

Abbreviation: PDCs, patient‐derived HCC cell lines.

### 3.2 Molecular targeting agents inhibit the intrahepatic growth of HCC cells

**Figure 3:**
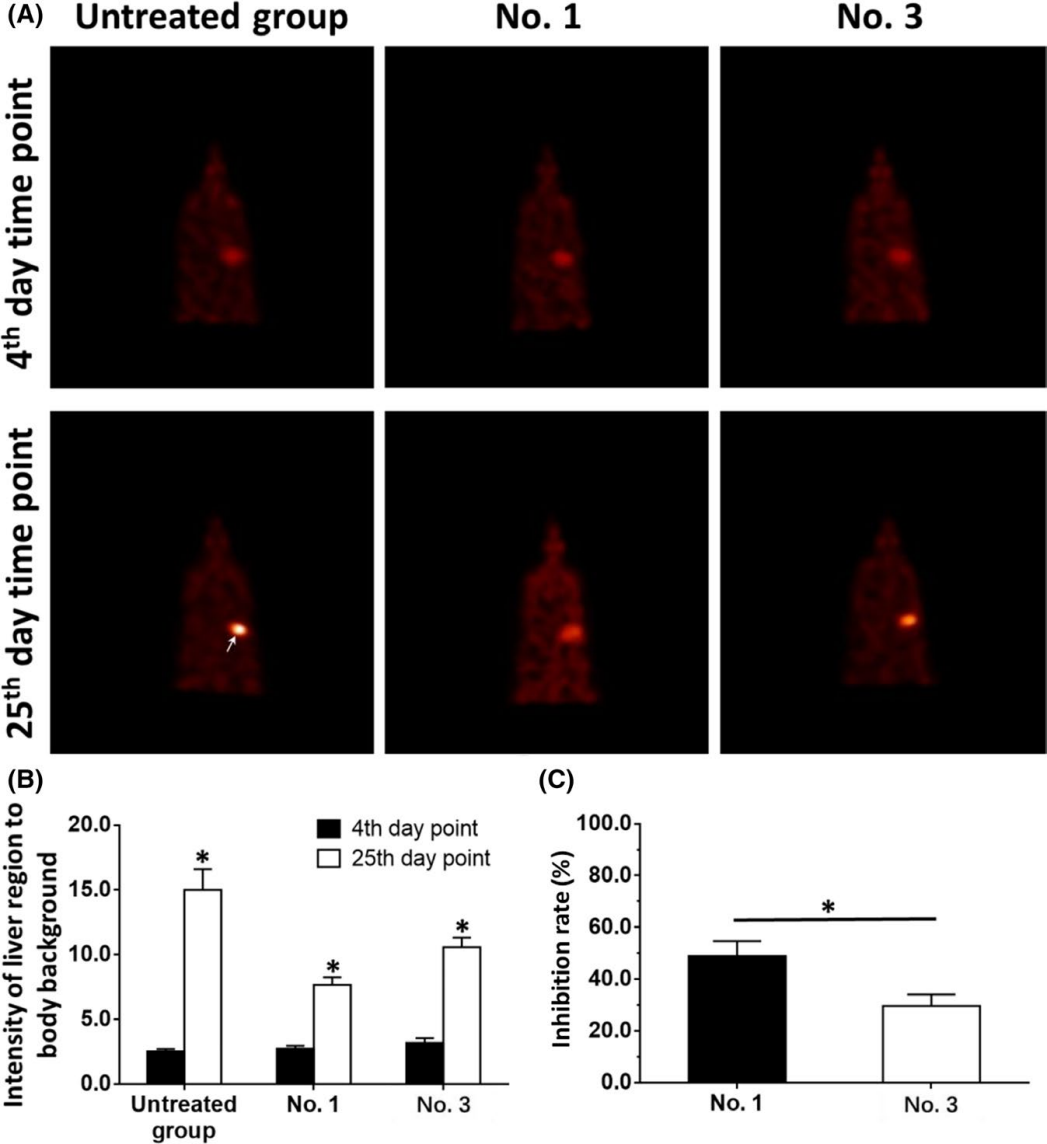
The inhibitory effect of sorafenib on the intrahepatic growth of patient‐derived HCC cell lines via MicroPET screening. Patient‐derived tissues containing HCC cells were injected into nude mice's liver organs to form intrahepatic tumors. The mice received 2 mg/kg dose of sorafenib via oral administration. Then, the mice received MicroPET screening and the images of MicroPET were quantitatively analyzed by image j software. The results are shown as images of MicroPET screening (A), intensity of 18F‐FDG absorbing in liver regions to body background (B) or inhibition rates (C). *P < .05

To examine the intrahepatic growth of HCC cells, microPET screening was used. The intrahepatic growth of HCC cells was examined by assessing the absorption of ^18^F‐FDG in PET images. The results are shown in Figure 3, Tables 3 and 4. Treatment using the molecular targeting agents decreased the absorption of ^18^F‐FDG in the liver region of nude mice. The effect of sorafenib, a typical molecular targeting agent, is shown in Figure 3 as a representative result and PDC No. 1 or PDC No. 3 had different sensitivities to sorafenib (Figure 3). The inhibition rates of the molecular targeting agents, sorafenib, regorafenib, lenvatinib, anlotinib, or apatinib in the five PDCs are shown in Table 3 (images) and Table 4 (livers). Among these molecularly targeted agents, lenvatinib had significantly higher antitumor activity against the subcutaneous growth of the HCC PDCs than the other four agents.

**Table 3:** Inhibition rates of agents on microPET images nude mice's liver region from the intrahepatic growth experiments of HCC cells

PDCs	No. 1	No. 2	No. 3	No. 4	No. 5
	Inhibition rates on microPET images (mean ± SD, %)				
Sorafenib	49.48 ± 3.45	33.94 ± 4.08	29.53 ± 2.88	35.30 ± 8.35	45.44 ± 7.96
Regorafenib	53.91 ± 4.80	29.69 ± 3.86	21.92 ± 5.48	24.86 ± 3.13	45.04 ± 6.75
Lenvatinib	56.95 ± 3.50	43.04 ± 6.28	36.37 ± 5.51	29.39 ± 4.31	44.41 ± 4.78
Anlotinib	36.28 ± 4.72	29.00 ± 2.88	23.83 ± 3.87	27.83 ± 5.23	27.36 ± 3.88
Apatinib	46.13 ± 5.45	37.20 ± 3.88	31.37 ± 3.19	40.50 ± 5.57	27.46 ± 3.84

Abbreviation: PDCs, patient‐derived HCC cell lines.

**Table 4:** Inhibition rates of agents on lesions formed by HCC cells in liver organs

PDCs	No. 1	No. 2	No. 3	No. 4	No. 5
	Inhibition rates on lesions in liver organs (mean ± SD, %)				
Sorafenib	45.22 ± 8.45	31.12 ± 6.59	23.34 ± 9.66	25.32 ± 2.16	25.88 ± 2.41
Regorafenib	45.53 ± 3.38	29.36 ± 7.11	15.15 ± 2.86	23.92 ± 4.40	36.03 ± 3.17
Lenvatinib	54.92 ± 3.54	41.15 ± 6.90	33.44 ± 5.92	26.09 ± 4.27	38.35 ± 3.84
Anlotinib	36.32 ± 4.36	26.02 ± 3.92	14.19 ± 1.61	23.06 ± 3.76	25.10 ± 4.29
Apatinib	37.01 ± 3.98	33.54 ± 2.96	27.55 ± 7.95	33.45 ± 3.08	27.55 ± 3.71

Abbreviation: PDCs, patient‐derived HCC cell lines.

**Figure 4:**
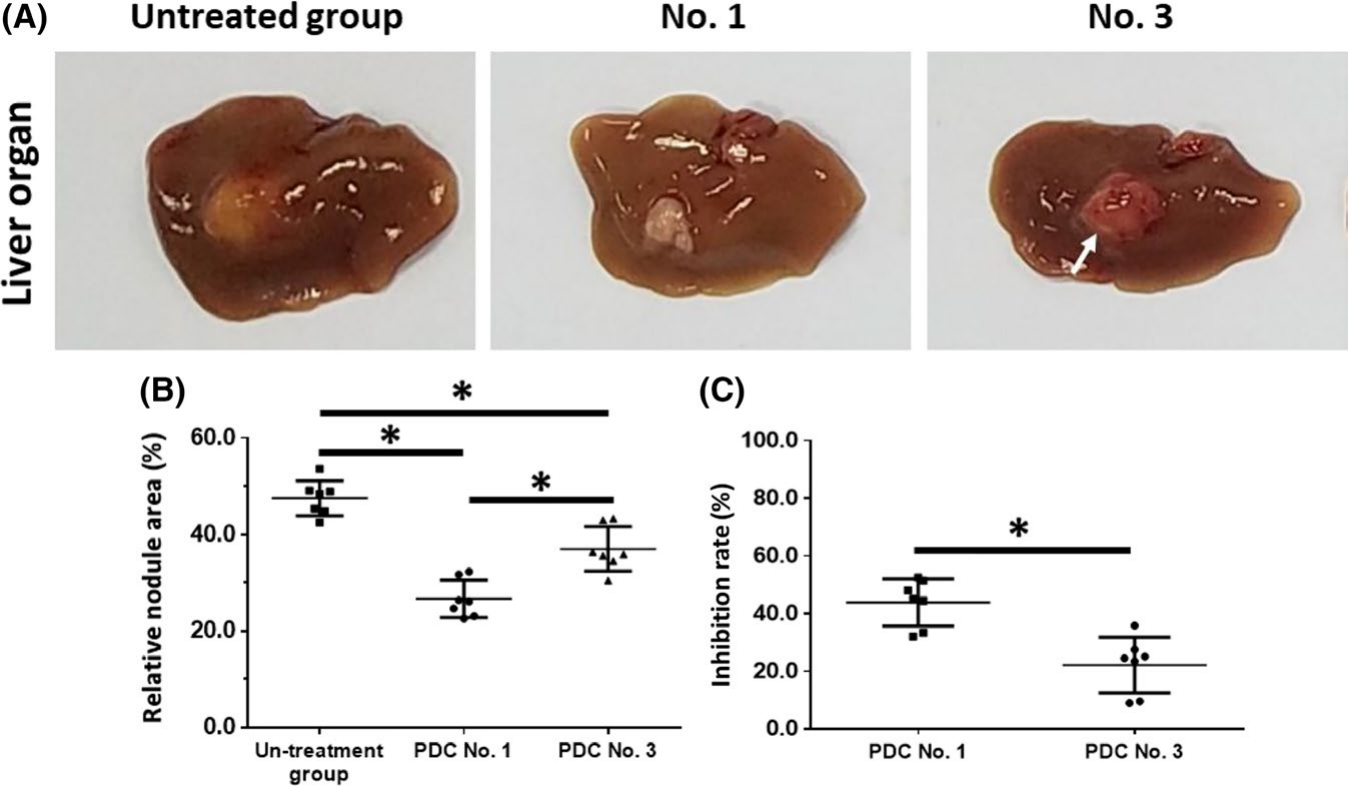
The inhibitory effect of sorafenib on the intrahepatic growth of patient‐derived HCC cell lines by collecting liver organs with lesions formed by HCC cells. Patient‐derived tissues containing HCC cells were injected into nude mice's liver organs to form intrahepatic tumors. Mice received 2 mg/kg dose of sorafenib via oral administration. After the mice received MicroPET screening, the liver organs of nude mice were collected and the images were quantitatively analyzed by image j software. The results are shown as images of liver organs with lesions formed by HCC cells (A), relative area of lesions in liver organs (B) or inhibition rates calculated by relative area of lesions in liver organs (C). *P < .05

The injection of HCC tumor tissue microblocks derived from patients into nude mouse livers resulted in the formation of nodules or lesions. The livers of the nude mice were harvested. The intrahepatic growth of the HCC cells is shown in Figure 4. Oral administration of the molecular targeting agent sorafenib inhibited the intrahepatic growth of HCC cells in nude mice (Figure 4, Tables 3 and 4). The effect of sorafenib, a typical molecular targeting agent, is shown in Figure 4 as a representative result and PDC No. 1 or PDC No. 3 had different sensitivities to sorafenib (Figure 4). The inhibition rates of the molecular targeting agents, sorafenib, regorafenib, lenvatinib, anlotinib, or apatinib in the five PDCs in terms of invasion in the liver are shown in Tables 3 and 4. Lenvatinib had significantly higher antitumor activity against the intrahepatic growth of HCC PDCs compared to the other four agents.

### 3.3 Molecular targeting agents inhibit the invasion of HCC cells into the liver

**Figure 5:**
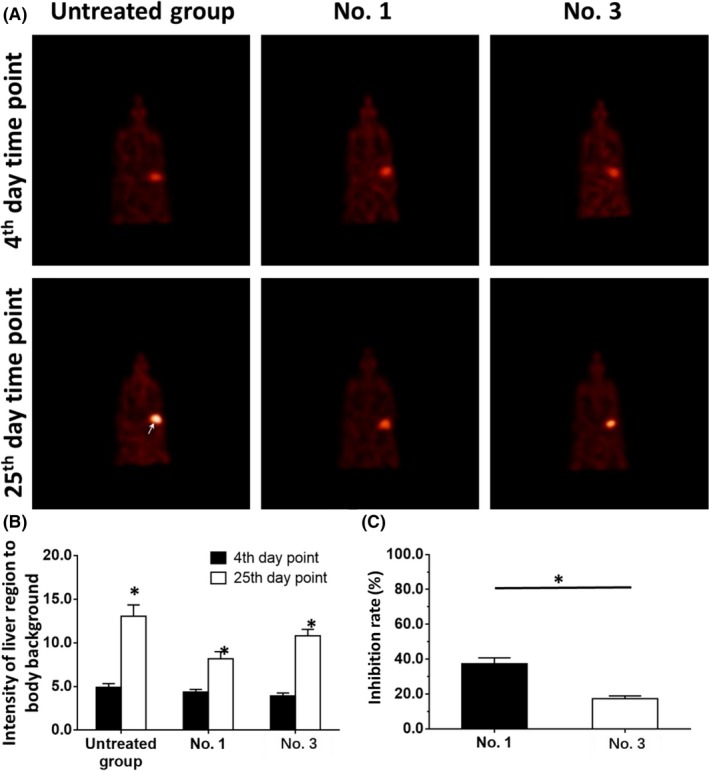
The inhibitory effect of sorafenib on the intrahepatic growth of patient‐derived HCC cell lines via MicroPET screening. Patient‐derived tissues containing HCC cells were mixed with hydrogel to form a hydrogel droplet with tumor tissues. Then, the hydrogel droplet with tumor tissues was adhered onto the surface of nude mice's liver organs to form the intrahepatic invasion model. The mice received 2 mg/kg dose of sorafenib via oral administration. Then, the mice received MicroPET screening and the images of MicroPET are quantitatively analyzed by image j software. The results are shown as images of MicroPET screening (A), intensity of 18F‐FDG absorbing in liver regions to body background (B) or inhibition rates (C). *P < .05

To further examine the effect of the molecular targeting agents, the invasion of HCC cells into the liver was examined. The intrahepatic invasion of HCC cells can be detected by observing the absorption of ^18^F‐FDG using microPET screening. The results are shown in Figure 5, Tables 5 and 6, and treatment using molecular targeting agents decreased ^18^F‐FDG absorption in the liver region of nude mice. The effect of sorafenib, a typical molecular targeting agent, is shown in Figure 5 as a representative result and PDC No. 1 and PDC No. 3 had different sensitivities to sorafenib (Figure 5). The inhibition rates of the molecular targeting agents, sorafenib, regorafenib, lenvatinib, anlotinib, or apatinib in the five PDCs are shown in Tables 5 and 6. Among these molecularly targeted agents, lenvatinib had significantly higher antitumor activity against the subcutaneous growth of the HCC PDCs than the other four agents.

**Table 5:** Inhibition rates of agents on microPET images nude mice's liver region from the invasive growth experiments of HCC cells

PDCs	No. 1	No. 2	No. 3	No. 4	No. 5
	Inhibition rates on microPET images (mean ± SD, %)				
Sorafenib	39.13 ± 7.74	26.07 ± 3.78	19.71 ± 3.22	23.62 ± 4.54	24.29 ± 5.31
Regorafenib	36.77 ± 6.91	24.30 ± 3.80	13.72 ± 2.60	19.43 ± 3.82	27.79 ± 6.02
Lenvatinib	39.73 ± 6.65	33.99 ± 6.49	28.96 ± 4.28	25.27 ± 2.96	31.93 ± 8.74
Anlotinib	29.13 ± 4.20	24.83 ± 5.09	14.00 ± 2.89	18.14 ± 2.58	20.32 ± 2.69
Apatinib	34.02 ± 4.01	30.49 ± 4.58	24.10 ± 4.63	27.78 ± 6.42	24.98 ± 2.83

Abbreviation: PDCs, patient‐derived HCC cell lines.

**Table 6:** Inhibition rates of agents on the intrahepatic invasion of HCC cells into nude mice's liver organs

PDCs	No. 1	No. 2	No. 3	No. 4	No. 5
	Inhibition rates on lesions in liver organs (mean ± SD, %)				
Sorafenib	50.26 ± 10.55	37.61 ± 6.24	26.49 ± 4.56	30.70 ± 7.67	32.24 ± 6.16
Regorafenib	53.15 ± 10.75	29.65 ± 4.46	18.58 ± 3.57	25.00 ± 4.18	40.69 ± 7.78
Lenvatinib	60.33 ± 11.34	42.01 ± 7.07	39.64 ± 7.08	35.01 ± 4.91	48.62 ± 8.34
Anlotinib	39.22 ± 5.66	32.15 ± 5.74	18.43 ± 3.91	27.65 ± 4.66	28.24 ± 2.45
Apatinib	43.82 ± 8.17	39.41 ± 6.28	34.87 ± 3.40	39.59 ± 5.85	31.94 ± 4.45

Abbreviation: PDCs, patient‐derived HCC cell lines.

**Figure 6:**
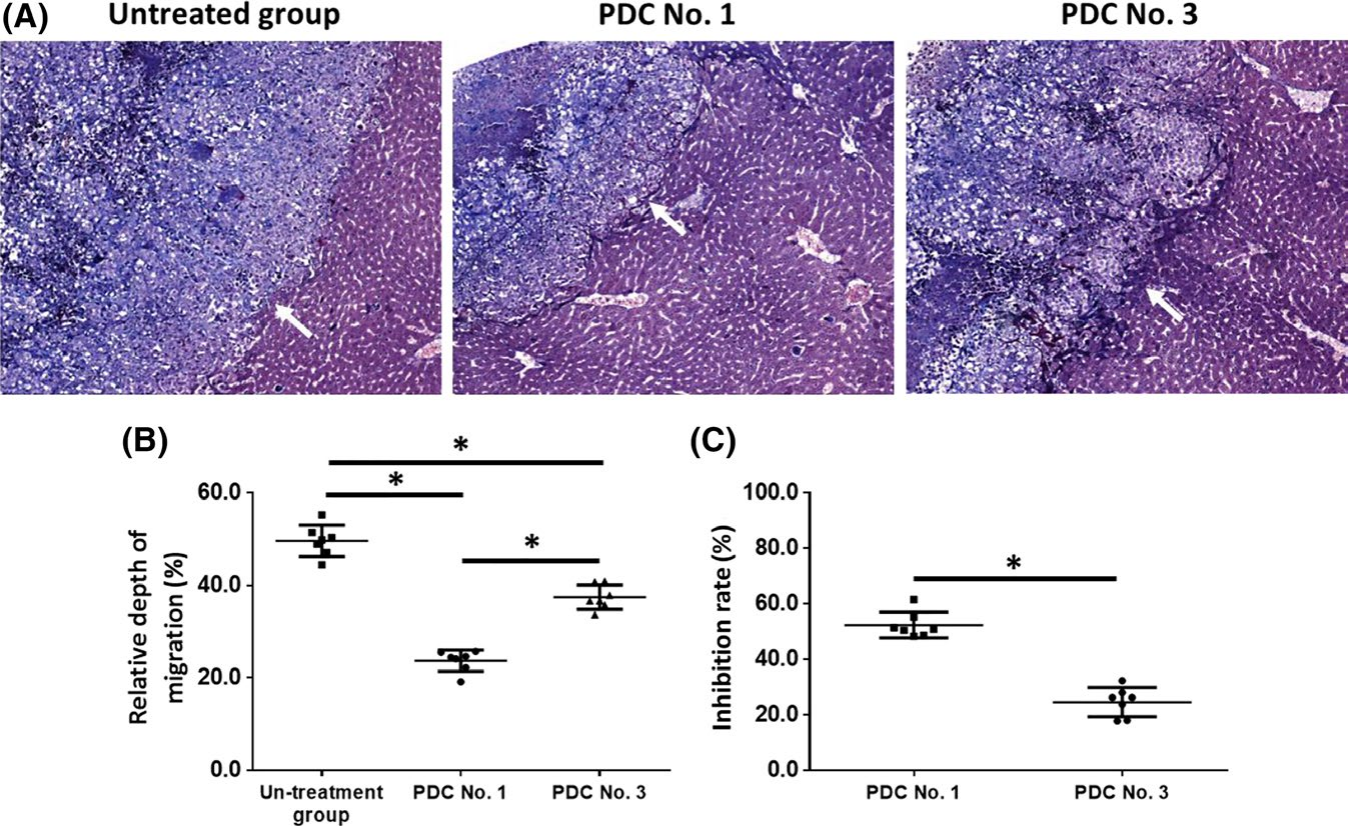
The inhibitory effect of sorafenib on the intrahepatic growth of patient‐derived HCC cell lines via MicroPET screening. Patient‐derived tissues containing HCC cells were mixed with hydrogel to form a hydrogel droplet with tumor tissues. Then, the hydrogel droplet with tumor tissues was adhered onto the surface of nude mice's liver organs to form the intrahepatic invasion model. Mice received 2 mg/kg dose of sorafenib via oral administration. After the mice received MicroPET screening, the liver organs of nude mice were harvested for H&E staining. The images of H&E are quantitatively analyzed by image j software. The results are shown as images of H&E staining (A), the relative depth of HCC cells invading into liver organs (B) or inhibition rates calculated based on (C). *P < .05

As shown in Figure 6, the invasion of the HCC cells in the liver was examined using H&E staining. Oral administration of the molecular targeting agents inhibited the subcutaneous growth of the HCC cells in nude mice (Figure 6, Tables 5 and 6). The effect of sorafenib, a typical molecular targeting agent, is shown in Figure 6 as a representative result and PDC No. 1 and PDC No. 3 had different sensitivities to sorafenib. The inhibition rates of the molecular targeting agents, sorafenib, regorafenib, lenvatinib, anlotinib or apatinib in the five PDCs in terms of invasion in the liver are shown in Tables 5 and 6. Lenvatinib had significantly higher antitumor activity against the subcutaneous growth of HCC PDCs compared to the other four agents.

## 4 DISCUSSION

Hepatitis viruses, such as HBV, have a very high infection rate in China and related East‐Asian regions.^1^, ^16^ Recently, more than 80 million people in China were reported to be infected with HBV or to suffer with various chronic liver diseases related to HBV.^1^, ^2^, ^16^, ^17^ Although many advances have been achieved in terms of antiviral treatments for HBV, patients with HBV‐related chronic liver disease may still have disease progression and eventually suffer from HCC.^16^, ^17^, ^18^ This means that HCC is a serious health threat and it also poses a great challenge for the public health system. Unfortunately, because of current clinical diagnostic and treatment strategies, most HCC patients have an advanced stage of HCC at initial diagnosis and are unsuitable for surgery or liver transplantation.^19^ Because advanced HCC has MDR characteristics for traditional cytotoxic chemotherapeutic drugs, existing antitumor treatment strategies mainly include various molecularly targeted drugs, such as sorafenib.^4^ Sorafenib was approved by the U.S. food and drug administration (FDA) in 2007 for the treatment of advanced HCC, and sorafenib drug resistance has been reported.^6^ To solve this problem, it is necessary to elucidate the molecular mechanism of sorafenib resistance. Despite the progress made in related research, the molecular mechanism of sorafenib resistance is still not very clear, and there is no convincing indicator for the clinical outcomes of patients undergoing treatment with sorafenib.^6^ Therefore, using a variety of pharmacological and experimental techniques to examine the sensitivity of tumor cells to molecularly targeted drugs in patient tissues is important to determine whether a patient is eligible for treatment before receiving molecular targeted therapy.^20^ In this study, patient‐derived HCC tumor tissues were collected and prepared into tissue microblocks which were then mixed with hydrogel droplets. Next, the hydrogel droplets containing the HCC tissue microblocks were attached to the surface of the liver in nude mice, so that the HCC cells could invade the liver. Molecular targeting therapies were used to treat the animals, and the inhibition rate of the HCC cell invasion reflected the sensitivity of the patients to the molecularly targeted drugs. In the future, the relationship between the inhibition rate of molecularly targeted agents in terms of HCC cell intrahepatic invasion and clinical prognosis will be determined. Moreover, our study achieved HCC tumor tissue by liver puncture at the time of initial diagnosis, and then could predict the sensitivity of HCC cells to molecular targeting agents such as Sorafenib. Whether patients receiving molecular targeted therapy or not is affected by many factors. At this stage, patients have not yet received molecular targeted therapy and there are no clinical data on the prognosis of patients. But the relevant clinical data of patients can be collected during the clinical treatment. Combined with the patient's survival status and follow‐up information, it is more helpful to confirm the results of this study.

Molecular targeting therapies represent the foremost strategy for advanced HCC treatment. Sorafenib has been used clinically for a long time and there are reports of related drug resistance.^6^ Recently, some new molecularly targeted drugs have been approved for HCC treatment: lenvatinib was approved as a first‐line treatment for advanced HCC; regorafenib was approved as a second‐line treatment for advanced HCC.^21^, ^22^ Lenvatinib and regorafenib have been in clinical use for a short time, and there may be reports of drug resistance in the future. These drugs not only inhibit the proliferation of HCC cells, but also delay disease progression.^21^, ^22^ Moreover, these agents can directly inhibit the metastasis and invasion of HCC cells by acting on RTK (receptor protein tyrosine kinase) and other MAPK signaling pathways such as Ras.^21^, ^22^ This study examined the in vivo invasion (intrahepatic invasion) of HCC cells in nude mice, which not only allows a determination of the antitumor activity of molecular targeting agents, but also has important implications for related research. Sorafenib has been widely used in clinical treatment and regorafenib or lenvatinib have been newly approved by the FDA.^23^, ^24^, ^25^, ^26^ In addition to these three agents, anlotinib and apatinib have been developed by Chinese manufacturers.^27^, ^28^, ^29^, ^30^, ^31^ Anlotinib has been used for NSCLC (nonsmall cell lung cancer) treatment and apatinib is used for gastric carcinoma. Recently, the antitumor effect of anlotinib and apatinib has been reported.^11^, ^32^, ^33^ The current results have extended our knowledge about anlotinib or apatinib and have indicated that among the tested molecular targeting agents, lenvatinib has significantly higher antitumor activity against the growth of HCC PDCs.

The subcutaneous tumor formation of HCC cells in nude mice is a commonly used model in oncology research, but the subcutaneous microenvironment is significantly different from the liver microenvironment. Moreover, traditionally, the detection of HCC cell metastasis and invasion is performed using transwell experiments. Although transwell experiments can mimic the metastasis and invasion of HCC cells, they are performed in vitro, and are not as informative as animal models. Ji et al and Liang et al used a tail vein injection to inoculate tumor cells and mimic metastasis.^34^, ^35^ Tumor cells injected into the tail vein will pass through the inferior vena cava and reach the lungs through the pulmonary artery. Because of the dense capillaries in the lungs, the tumor cells are retained. Our previous work established another approach: injection of HCC cells into the liver tissue via the hepatic portal vein, which results in multiple and diffuse tumor lesions that mimic the highly aggressive features of advanced HCC.^13^ In this work, the adhesion of HCC tumor tissues onto the surface of the liver in nude mice was used to mimic the in vivo invasion of HCC cells. The results showed that HCC cells can penetrate the surface of the liver in nude mice, and invade and destroy underlying tissues. Treatment using molecular targeting agents inhibited the invasion of HCC cells into the liver. This model can directly reflect the invasion of HCC cells and enable quantitative research. Wei et al and Meng et al performed similar research using cell lines.^36^, ^37^, ^38^ In this study, the use of patient‐derived HCC tissue was more clinically meaningful compared with the use of cell lines in other studies. HCC cells in solid tumor tissues grow in a 3‐D pattern and the influence of the ECM (extra‐cellular matrix) modulates the behavior and features of solid tumors. The use of patient‐derived tumor tissues reflects patient features and also maintains the ECM of the clinical samples.

## CONFLICT OF INTEREST

None.

## AUTHOR CONTRIBUTIONS

All the listed authors meet the requirements for authorship. JH and RSL conceived and designed the experiments; HWS and FF performed the experiments and wrote the main manuscript text; HX, YTC and XJL performed the animal experiments; QTJ, RCY and ZJW performed the molecular biology experiment and analyzed the data. All authors have read and approved the manuscript.
