# Supplementary figures and images for: Quantitative examination of the inhibitory activation of molecular targeting agents in hepatocellular carcinoma patient‐derived cell invasion via a novel in vivo tumor model
Source: Animal Model Exp Med. 2019 Sep 27;2(4):259–68. doi: 10.1002/ame2.12085 (PMC6930997; doi:10.1002/ame2.12085)

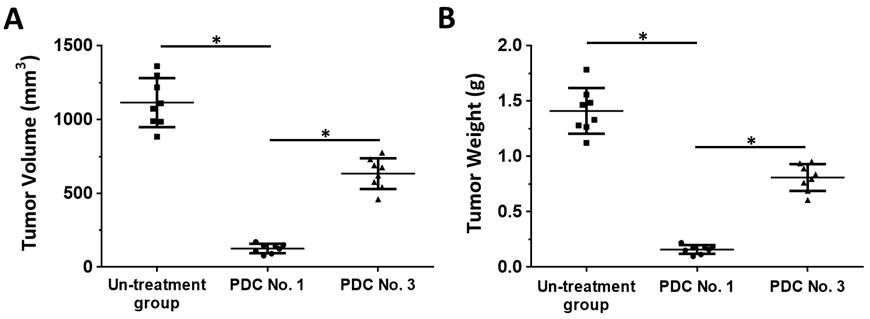

Supplement: Supplementary file 1 [file AME2-2-259-s001.jpg]
