# Supplementary material for: Quantitative examination of the inhibitory activation of molecular targeting agents in hepatocellular carcinoma patient‐derived cell invasion via a novel in vivo tumor model
Source: Animal Model Exp Med. 2019 Sep 27;2(4):259–68. doi: 10.1002/ame2.12085 (PMC6930997; doi:10.1002/ame2.12085)
Supplement: Supplementary file 2 [file AME2-2-259-s002.docx]

**SUPPLEMENTAL FIGURE 1** The antitumor effect of sorafenb on patients-derived HCC cell lines. Patients derived tissues containing HCC cells were injected into nude mice to form subcutaneous tumors. Mice were received 2mg/kg dose of sorafenib via oral administration. Then, tumor tissues were collected and the tumor volumes or tumor weights were examined. The results were shown as images of subcutaneous tumors (A), tumor volumes (B) or tumor weights (C). ^*^*P* < 0.05
